# Pre-diagnostic C-reactive protein concentrations, *CRP* genetic variation and mortality among individuals with colorectal cancer in Western European populations

**DOI:** 10.1186/s12885-022-09778-9

**Published:** 2022-06-24

**Authors:** Katharina Nimptsch, Krasimira Aleksandrova, Veronika Fedirko, Mazda Jenab, Marc J. Gunter, Peter D. Siersema, Kana Wu, Verena Katzke, Rudolf Kaaks, Salvatore Panico, Domenico Palli, Anne M May, Sabina Sieri, Bas Bueno-de-Mesquita, Karina Standahl, Maria-Jose Sánchez, Aurora Perez-Cornago, Anja Olsen, Anne Tjønneland, Catalina Bonet Bonet, Christina C. Dahm, María-Dolores Chirlaque, Valentina Fiano, Rosario Tumino, Aurelio Barricarte Gurrea, Marie-Christine Boutron-Ruault, Florence Menegaux, Gianluca Severi, Bethany van Guelpen, Young-Ae Lee, Tobias Pischon

**Affiliations:** 1 Molecular Epidemiology Research Group, Max Delbrück Center for Molecular Medicine in the Helmholtz Association (MDC), Berlin, Germany; 2 Department of Nutrition, Harvard T.H. Chan School of Public Health, Boston, MA USA; 3 Department Epidemiological Methods and Etiological Research, Leibniz Institute for Prevention Research and Epidemiology, Bremen, Germany; 4 Faculty of Human and Health Sciences, University of Bremen, Bremen, Germany; 5 Department of Epidemiology, University of Texas M. D. Anderson Cancer Center, Houston, TX USA; 6 Department of Epidemiology, Rollins School of Public Health, Emory University, Atlanta, GA USA; 7 International Agency for Research on Cancer (IARC-WHO), Lyon, France; 8 Department of Gastroenterology and Hepatology, Radboud university medical center, Nijmegen, The Netherlands; 9 Department of Cancer Epidemiology, German Cancer Research Center (DKFZ), Heidelberg, Germany; 10 Dipartimento di Medicina Clinica e Chirurgia, Federico II University, Naples, Italy; 11 Cancer Risk Factors and Life-Style Epidemiology Unit, Institute for Cancer Research, Prevention and Clinical Network - ISPRO, Florence, Italy; 12 Julius Center for Health Sciences and Primary Care, University Medical Center Utrecht, Utrecht University, Utrecht, The Netherlands; 13 Epidemiology and Prevention Unit, Fondazione IRCCS Istituto Nazionale dei Tumori di Milano, Milano, Italy; 14 Centre for Nutrition, Prevention and Health Services, National Institute for Public Health and the Environment, Bilthoven, The Netherlands; 15 Department of Community Medicine, Faculty of Health Sciences, UiT The Arctic University of Norway, Tromsø, Norway; 16 Escuela Andaluza de Salud Pública (EASP), Granada, Spain; 17 Instituto de Investigación Biosanitaria ibs.GRANADA, Granada, Spain; 18 Centro de Investigación Biomédica en Red de Epidemiología y Salud Pública (CIBERESP), Madrid, Spain; 19 Department of Preventive Medicine and Public Health, University of Granada, Granada, Spain; 20 Cancer Epidemiology Unit, Nuffield Department of Population Health, University of Oxford, Oxford, UK; 21 Danish Cancer Society Research Center, Copenhagen, Denmark; 22 Department of Public Health, University of Århus, Århus, Denmark; 23 Department of Public Health, University of Copenhagen, Copenhagen, Denmark; 24 Unit of Nutrition and Cancer, Catalan Institute of Oncology – ICO, Barcelona, Spain; 25 Nutrition and Cancer Group, Bellvitge Biomedical Research Institute – IDIBELL, Barcelona, Spain; 26 L’Hospitalet de Llobregat, Barcelona, Spain; 27 Department of Epidemiology, Regional Health Council, IMIB-Arrixaca, Murcia University, Murcia, Spain; 28 Cancer Epidemiology Unit, Department of Medical Sciences, University of Turin, Turin, Italy; 29 Cancer Registry and Histopathology Department Provincial Health Authority (ASP 7), Ragusa, Italy; 30 Navarra Public Health Institute, Pamplona, Spain; 31 Navarra Institute for Health Research (IdiSNA), Pamplona, Spain; 32 Paris-Saclay University, UVSQ, Gustave Roussy, “Exposome and Heredity” team, CESP UMR1018, Villejuif, Inserm France; 33 Department of Statistics, Computer Science and Applications “G. Parenti” (DISIA), University of Florence, Florence, Italy; 34 Department of Radiation Sciences, Oncology, Umeå University, Umeå, Sweden; 35 Wallenberg Centre for Molecular Medicine, Umeå University, Umeå, Sweden; 36 Genetics of Allergic Disease Research Group, Max Delbrück Center for Molecular Medicine in the Helmholtz Association (MDC), Berlin, Germany; 37 Pediatric Allergy Experimental and Clinical Research Center, Charité Campus Buch, Berlin, Germany; 38 Charité - Universitaetsmedizin Berlin, Corporate Member of Freie Universitaet Berlin, Humboldt-Universitaet zu Berlin, Berlin, Germany; 39 Max-Delbrueck-Center for Molecular Medicine in the Helmholtz Association (MDC), Biobank Technology Platform, Berlin, Germany; 40 Core Facility Biobank, Berlin Institute of Health at Charité - Universitätsmedizin Berlin, Berlin, Germany

## Abstract

**Background:**

The role of elevated pre-diagnostic C-reactive protein (CRP) concentrations on mortality in individuals with colorectal cancer (CRC) remains unclear.

**Methods:**

We investigated the association between pre-diagnostic high-sensitivity CRP concentrations and *CRP* genetic variation associated with circulating CRP and CRC-specific and all-cause mortality based on data from 1,235 individuals with CRC within the European Prospective Investigation into Cancer and Nutrition cohort using multivariable-adjusted Cox proportional hazards regression.

**Results:**

During a median follow-up of 9.3 years, 455 CRC-specific deaths were recorded, out of 590 deaths from all causes. Pre-diagnostic CRP concentrations were not associated with CRC-specific (hazard ratio, HR highest versus lowest quintile 0.92, 95% confidence interval, CI 0.66, 1.28) or all-cause mortality (HR 0.91, 95% CI 0.68, 1.21). Genetic predisposition to higher CRP (weighted score based on alleles of four *CRP* SNPs associated with higher circulating CRP) was not significantly associated with CRC-specific mortality (HR per *CRP*-score unit 0.95, 95% CI 0.86, 1.05) or all-cause mortality (HR 0.98, 95% CI 0.90, 1.07). Among four investigated *CRP* genetic variants, only SNP rs1205 was significantly associated with CRC-specific (comparing the CT and CC genotypes with TT genotype, HR 0.54, 95% CI 0.35, 0.83 and HR 0.58, 95% CI 0.38, 0.88, respectively) and all-cause mortality (HR 0.58, 95% CI 0.40, 0.85 and 0.64, 95% CI 0.44, 0.92, respectively).

**Conclusions:**

The results of this prospective cohort study do not support a role of pre-diagnostic CRP concentrations on mortality in individuals with CRC. The observed associations with rs1205 deserve further scientific attention.

**Supplementary Information:**

The online version contains supplementary material available at 10.1186/s12885-022-09778-9.

## Introduction

Colorectal cancer is the third most commonly diagnosed cancer worldwide and the second leading cause of cancer death, with an estimated number of almost one million deaths worldwide in 2020 [1]. Plausible evidence points to chronic inflammation playing an important role in colorectal carcinogenesis, as it has been consistently observed that individuals with chronic inflammatory bowel disease have a higher risk of colorectal cancer (CRC) [2, 3], whereas the regular use of anti-inflammatory drugs has been associated with lower CRC risk [4, 5]. Chronic low-grade inflammation may facilitate carcinogenic processes through promoting tumor cell proliferation, cell survival and migration [6] and there is also evidence that systemic inflammation is linked to local tissue-specific inflammation in the colorectal mucosa [7]. Higher concentrations of the inflammatory biomarker C-reactive protein (CRP) have been associated with a moderately higher risk of colorectal cancer (CRC) in a meta-analysis of eighteen prospective studies [8], although significant heterogeneity was observed across individual studies. The positive association with pre-diagnostic CRP was observed for colon but not for rectal cancer, and among men but not women. In the most recent investigation, a population-based nested case-control study from Northern Sweden, CRP concentrations were not related to subsequent risk of CRC, regardless of CRC location, stage or molecular subtype [9]. In the European Prospective Investigation into Cancer and Nutrition (EPIC), we previously observed that elevated circulating CRP concentrations were associated with a higher risk of colon but not rectal cancer and a higher risk of colon cancer was particularly observed in men but not in women [10]. In a subsequent analysis, we investigated the association of *CRP* genetic variants with CRP concentrations and CRC risk in EPIC. Of five *CRP* tagging SNPs, four (rs1205, rs1800947, rs1130864 and rs3093077) were significantly associated with CRP concentrations in control participants and were incorporated in a genetic *CRP*-score which was associated with 13% higher CRP concentrations per allele count, explaining 2% of inter-individual variation in CRP concentrations. We observed that the minor alleles of two *CRP* SNPs (rs1205 and rs1130864) as well as the genetic *CRP*-score were associated with higher CRC risk, slightly more pronounced for colon compared to rectal cancer [11]. One large Mendelian randomization study (30,480 CRC cases, 22, 844 controls) did not confirm that genetically determined (including CRP-associated SNPs both inside and outside the *CRP* gene) higher CRP concentrations are associated with higher CRC risk [12], while another recent Mendelian randomization study (10,142 women of whom 734 developed CRC) found associations between genetically determined CRP and CRC risk in subgroups of lifestyle factors, i.e. in non-viscerally obese and individuals with high-fat diet [13]. Although these findings shed doubt on the potential causal association between high CRP and higher CRC risk, the question whether circulating CRP may be associated with CRC mortality remains unclear. Higher CRP concentrations at or after diagnosis (pre-treatment) have been reported to be associated with poor survival in CRC patients in a systematic review including 12 retrospective prognostic studies including 1705 patients in total [14], but in these studies, circulating CRP may reflect the presence of the tumor, which leads to both localized and systemic inflammatory response [6, 15]. Circulating CRP level after diagnosis is also part of the modified Glasgow Prognostic score, which is clinically used as inflammation-based prognostic parameter in colorectal cancer patients [16]. In contrast, there is little evidence on whether pre-diagnostic CRP concentrations play a significant role in survival outcomes of individuals with CRC. So far, two prospective studies explored the association between pre-diagnostic circulating CRP and survival in persons with CRC reporting null findings [17, 18], but one [18] had small sample size (*n* = 173 CRC cases) and the other [17] did not use a high-sensitivity CRP assay, precluding the detection of low-grade inflammation. In terms of *CRP* genetic variation leading to genetic predisposition to lifelong elevated CRP concentrations, two previous studies explored various *CRP* genetic variants in relation to CRC survival reporting conflicting results [19, 20]. In addition, a recent large Mendelian Randomization analysis using a genetic risk score based on 52 genetic variants associated with CRP levels identified from genome-wide association studies did not support a causal effect of circulating CRP concentrations on CRC-specific survival [21].

The aim of our study was to take advantage of the availability of both measured CRP concentrations and *CRP* genetic variants in EPIC to assess the association between pre-diagnostic CRP concentrations as well as *CRP* genetic variation associated with higher circulating CRP levels with CRC-specific and all-cause mortality in individuals with CRC.

## Methods

### Study population and data collection procedures

 This study comprises participants of the European Prospective Investigation into Cancer and Nutrition (EPIC) diagnosed with CRC post-enrollment. EPIC is a multicenter prospective cohort study, including 521,448 participants from 10 Western European countries (Denmark, France, Germany, Greece, Italy, the Netherlands, Norway, Spain, Sweden, and United Kingdom), who were between 25 and 70 years old at enrollment between 1992 and 2000 [22]. Information on participants’ sociodemographic and lifestyle characteristics as well as personal and medical history was collected with questionnaires at recruitment, and anthropometric measurements and blood samples were taken [22–24]. Blood samples were separated into 0.5 ml fractions (plasma, serum, red blood cells and buffy coat for DNA extraction), placed into straws and stored centrally at the International Agency for Research on Cancer (IARC) in Lyon, France. Samples were kept at -196 °C in liquid nitrogen for all EPIC countries except Denmark (1.0 ml aliquots stored at -150 °C under nitrogen vapor) and Sweden (aliquots stored in freezers at -80 °C), where aliquots were stored locally. Written informed consent was obtained from all participants. The EPIC study was ethically approved by the review board of the IARC as well as the local review boards pertaining to the participating institutions in the respective countries. All methods were carried out in accordance with relevant guidelines and regulations’ or the ‘Declaration of Helsinki’.

### Cancer incidence follow-up

Incident cancer cases were identified through record linkage with regional cancer registries in most study centers (for the present analysis complete up to June 2003), whereas a combination of methods, including health insurance records, cancer and pathology registries, as well as active follow-up through direct contact with study participants or their next-of-kin was used in the study centers of France, Germany, Greece and Naples, Italy (complete up to June 2002).

### Vital status follow-up

Vital status (follow-up complete for 98.5%) was determined through record linkage with regional and/or national mortality registries, with the exception of France, Germany, and Greece where vital status was ascertained through follow-up procedures based on a combination of methods as described for the identification of cancer cases. For these centers, the end of follow-up was the last known date of contact or the date of death, whichever came first (last update between December 2006 and March 2015). For the study centers using record linkage, censoring dates of complete vital status follow-up were between December 2009 and December 2014. The 10th revision of the International Classification of Diseases, Injuries and Causes of Death (ICD-10) was used to code the underlying cause of death, as main outcome in the present study.

### Selection of CRC cases

The selection of CRC cases has been previously described in detail [25]. Eligible for this study were participants of the EPIC study who developed tumors of the colon (ICD-10 C18.0-C18.7), rectum (C19-C20) or tumors that were overlapping or unspecified (C18.8-C18.9) during the follow-up period. A total of 1,235 CRC cases with CRP measurement were included in the present analysis (no cases from EPIC Greece were included due to technical reasons and no cases from Norway because of low number of CRC cases in EPIC Norway). Data on genetic variation in *CRP* SNPs was available for 822 of these CRC cases (there were no CRC cases with available *CRP* SNPs but no CRP measurement). No DNA samples were available from Danish EPIC centers due to local technical and organizational issues.

### Measurement of CRP concentrations, *CRP* SNP selection and genotyping procedures

Serum CRP was determined using a high sensitivity assay in the same laboratory [10]. As previously described [11], five tagging SNPs were selected via HapMap 22/phaseII CePH applying stringent criteria (minor allele frequency > 5% and pairwise LD r^2^ ≥ 0.80) to cover variations in the *CRP* gene in populations of European descent as well as based on findings from a genome-wide association study that identified two *CRP* SNPs significantly associated with CRP concentrations [26]. The selected SNPs (rs1205, rs1800947, rs1130864, rs2808630, rs3093077) were genotyped with TaqMan methodology with genotype call rates > 99.2% for all assays.

### Statistical analysis

Characteristics of individuals who were diagnosed with colorectal cancer are presented across CRP quintiles as frequencies and proportions for categorical variables, as mean (SD) for continuous variables with approximate normal distribution and as median (25th and 75th percentile) for skewed variables. Diabetes at baseline was defined as either self-reported diabetes diagnosis or HbA1c ≥ 6.5%. Family history of CRC was defined as self-reported past diagnosis of CRC in a first-degree relative (mother, father, sister or brother).

We investigated the association between pre-diagnostic CRP concentrations and *CRP* SNPs and CRC-specific (primary endpoint) and all-cause mortality (secondary endpoint), using Cox proportional hazards regression models stratified by country with age at CRC diagnosis and event/censorship as underlying time scales to control for age. The date of death from CRC or all causes, respectively, was considered the event of interest and participants were censored at date of death from other causes (in the analyses of CRC-specific death), date of last known contact or the date at which vital status follow-up through record linkage was considered to be complete. The proportional hazards assumption was met as evaluated by including interactions of CRP concentrations and CRP SNPs, respectively, and survival time as time-dependent variables in the Cox models and evaluating their statistical significance [27]. We calculated multivariable hazard ratios (HRs) and 95% confidence intervals (CI) adjusting for age at diagnosis, sex, cancer stage and grade of tumor differentiation at diagnosis, location of tumor and year of diagnosis. Because lifestyle factors such as smoking status, body mass index and physical activity may influence circulating CRP and potentially also mortality after CRC diagnosis, they were included in the models investigating CRP concentrations and mortality in individuals with CRC as potentially confounding factors (for physical activity in MET-hours/week, *n* = 77 missing values were imputed with sex-specific median values). Pre-diagnostic CRP concentrations were analyzed continuously (primary analysis) as (naturally) log-transformed CRP divided by log 2, corresponding to a doubling in CRP on the original scale as well as in quintiles. In sensitivity analyses, we used established cut-offs for hsCRP (< 1, 1–3, > 3 mg/L) instead of quintiles as categorical variable. In addition, we investigated potential non-linear associations by adding a quadratic or cubic term of CRP to the model and examined whether this improved the model significantly using the likelihood ratio test.

We conducted subgroup analyses by sex, tumor location, tumor stage as well as BMI (< 25, 25-29.9, ≥ 30 kg/m^2^) and waist circumference (</≥88 cm in women, </≥102 cm in men) categories. In addition, to account for potential interaction between CRP and processed meat intake as previously observed in EPIC [10], we stratified models by red and processed meat intake using pre-defined cut-offs (</≥ 48.8 g/day red meat, </≥ 25.5 g/day processed meat). Tests for multiplicative interaction were performed by including a cross-product term of CRP as continuous variable (log-transformed divided by log 2) and the variable of interest and utilizing the Wald test to assess statistical significance. In sensitivity analyses we repeated the multivariable analyses for circulating CRP stratifying by time between recruitment (and blood collection) and CRC diagnosis,, excluding participants with CRP concentrations ≥ 10 mg/L, that may characterize acute inflammatory state (*n* = 99) as well as after exclusion of diabetics (*n* = 123) and individuals with family history of CRC (*n* = 27).

We investigated the association between *CRP* SNPs and circulating CRP (log-transformed) in individuals with CRC using univariate linear regression models, calculating the percentage difference in CRP on the original scale. For the analysis of the association between genetic predisposition to higher CRP concentrations and mortality in individuals with CRC, we created a weighted *CRP* allele score as previously described [11] by counting the alleles individually associated with higher CRP concentrations (score based on SNPs rs1205, rs180047, rs1130864 and rs3093077) and using their estimated coefficients from the linear regression as weights [28]. In addition to the *CRP*-score, the associations between individual *CRP* SNPs and CRC and all-cause mortality were investigated. Individual SNPs were coded by genotype, with the genotype associated with the lowest CRP concentrations as reference, as well as continuously according to the number of alleles associated with higher CRP concentrations (coded as 0,1,2).

We calculated the minimal detectable HRs with a power of 0.8 for the primary analysis (continuous exposure variables, CRC-specific death) and the given sample size using SAS proc power, assuming a two-sided test for a one-unit increase in either circulating CRP or weighted CRP-score in Cox proportional hazards regression. Based on these calculations, the minimal detectable HR for a doubling in circulating CRP (log-transformed divided by log 2, standard deviation 1.75) is 1.08. With the given sample size for the genetic analyses, the minimal detectable HR for a one-unit increase in *CRP*-score (standard deviation 1.36) with power 0.8 would be 1.14.

All statistical analyses were performed with SAS® Enterprise Guide® 7.15 (SAS Institute Inc., Cary, North Carolina, USA).

## Results

Among the 1,235 individuals with CRC included in our analysis, 590 deaths from all causes were recorded during the follow-up, of which 455 were due to colorectal cancer. Median follow-up time after CRC diagnosis was 9.3 years (25th percentile 1.9 years, 75th percentile 13.3 years). The median time difference between blood collection and CRC diagnosis was 3.9 years (25th percentile 2.2, 75th percentile 5.6).

Characteristics of the individuals with CRC by CRP quintiles are shown in Table 1. Age at diagnosis increased slightly across CRP quintiles, while no trends across quintiles were observed for percentage of women, or physical activity or family history of CRC. The proportion of current smokers was highest (29.8%) in the lowest CRP quintile and lower (between 21.2% and 27.1%) in the upper quintiles. As previously reported [10], mean BMI and waist circumference values increased across CRP quintiles. The proportion of diabetics at baseline increased across CRP quintiles.

**Table 1:** Characteristics of colorectal cancer patients by C-reactive protein (CRP) quintiles

		Quintile 1	Quintile 2	Quintile 3	Quintile 4	Quintile 5
N		248	245	249	247	246
CRP Quintile ranges, mg/L		0.20–0.73	0.74–1.81	1.82–3.31	3.32–5.57	≥ 5.58
CRP, mg/L, median (Q25, Q75)		0.34 (0.20, 0.47)	1.19 (0.95, 1.45)	2.53 (2.16, 2.87)	4.21 (3.65, 4.87)	8.86 ( 6.82–13.02)
Female sex, n (%)		131 (52.8)	111 (45.3)	113 (45.4)	133 (53.8)	138 (56.1)
Age at diagnosis, years, mean (SD)		61.0 (7.4)	62.3 (7.4)	62.4 (7.1)	63.3 (7.0)	63.1 (7.3)
Current smoking, n (%)		74 (29.8)	52 (21.2)	61 (24.5)	67 (27.1)	58 (23.6)
Physical activity (MET-hours/week), mean (SD)		83.6 (52.7)	85.7 (55.5)	81.7 (50.1)	85.2 (51.7)	86.5 (54.9)
Body mass index, kg/m^2^, mean (SD)		24.5 (3.4)	26.0 (3.6)	26.7 (3.8)	27.4 (4.1)	28.9 (5.3)
Waist circumference, cm, mean (SD)		84.1 (11.9)	88.9 (12.6)	90.8 (11.9)	92.3 (12.5)	95.9 (13.8)
Diabetes, n (%)		12 ( 5.0)	14 ( 5.9)	17 ( 6.9)	36 (14.7)	44 (18.6)
Family history of CRC, n (%)		6 ( 9.7)	4 ( 6.7)	4 ( 7.1)	6 ( 8.8)	7 (10.3)
Location of primary tumor, n (%)						
Colon		152 (61.3)	139 (56.7)	148 (59.4)	160 (64.8)	181 (73.6)
Rectum		96 (38.7)	106 (43.3)	101 (40.6)	87 (35.2)	65 (26.4)
Grade of differentiation, n (%)						
Well differentiated		16 (6.5)	11 (4.5)	15 (6.0)	15 (6.1)	14 (5.7)
Moderately differentiated		66 (26.6)	65 (26.5)	73 (29.3)	81 (32.8)	74 (30.1)
Poorly differentiated		14 (5.6)	21 (8.6)	17 (6.8)	9 (3.6)	20 (8.1)
Unknown		152 (61.3)	148 (60.4)	144 (57.8)	142 (57.5)	138 (56.1)
Stage, n (%)						
I		73 (29.4)	66 (26.9)	64 (25.7)	46 (18.6)	58 (23.6)
II		58 (23.4)	41 (16.7)	51 (20.5)	54 (21.9)	53 (21.5)
III		56 (22.6)	79 (32.2)	69 (27.7)	85 (34.4)	81 (32.9)
IV		25 (10.1)	20 (8.2)	34 (13.7)	34 (13.8)	32 (13.0)
Unknown		36 (14.5)	39 (15.9)	31 (12.4)	28 (11.3)	22 (8.9)

Higher pre-diagnostic CRP concentrations were not significantly associated with higher risk of mortality from CRC (Table 2). Comparing the highest with the lowest quintile of pre-diagnostic CRP a HR of 0.92 (95% CI 0.66, 1.28) was observed. Similarly, no association between CRP and all-cause mortality was observed (HR highest versus lowest CRP quintile 0.91, 95% CI 0.68, 1.21).

**Table 2:** Association between pre-diagnostic C-reactive protein (CRP) concentrations and colorectal cancer outcome

	CRC mortality			All-cause mortality		
	N event/N Total	HR	(95% CI)	N event/N Total	HR	(95% CI)
**Colorectal cancer**						
Quintile 1	91/248	1	Reference	112/248	1	Reference
Quintile 2	89/245	0.98	(0.71, 1.34)	114/245	0.94	(0.71, 1.24)
Quintile 3	87/249	0.80	(0.58, 1.10)	116/249	0.79	(0.60, 1.05)
Quintile 4	94/247	0.85	(0.62, 1.17)	124/247	0.84	(0.64, 1.11)
Quintile 5	94/246	0.92	(0.66, 1.28)	124/246	0.91	(0.68, 1.21)
p-trend			0.76			0.76
per doubling in CRP		0.95	(0.89, 1.01)		0.96	(0.91, 1.01)
**Colorectal cancer, men only**						
Quintile 1	39/117	1	Reference	53/117	1	Reference
Quintile 2	48/134	1.00	(0.64, 1.57)	65/134	0.93	(0.63, 1.36)
Quintile 3	52/136	0.87	(0.55, 1.37)	67/136	0.76	(0.51, 1.13)
Quintile 4	45/114	1.00	(0.62, 1.60)	63/114	0.94	(0.63, 1.41)
Quintile 5	42/108	0.99	(0.60, 1.61)	58/108	0.89	(0.58, 1.34)
p-trend			0.94			0.86
per doubling in CRP		0.98	(0.90, 1.08)		0.97	(0.89, 1.05)
**Colorectal cancer, women only**						
Quintile 1	52/131	1	Reference	59/131	1	Reference
Quintile 2	41/111	1.00	(0.62, 1.60)	49/111	0.98	(0.64, 1.50)
Quintile 3	35/113	0.74	(0.45, 1.21)	49/113	0.88	(0.57, 1.36)
Quintile 4	49/133	0.71	(0.45, 1.13)	61/133	0.74	(0.49, 1.12)
Quintile 5	52/138	0.77	(0.47, 1.25)	66/138	0.90	(0.59, 1.39)
p-trend			0.32			0.68
per doubling in CRP		0.91	(0.83, 1.00)		0.94	(0.87, 1.02)
p-interaction by sex			0.18			0.31
**Colon cancer**						
Quintile 1	57/152	1	Reference	71/152	1	Reference
Quintile 2	45/139	0.88	(0.57, 1.36)	59/139	0.82	(0.56, 1.19)
Quintile 3	54/148	0.83	(0.54, 1.25)	75/148	0.80	(0.56, 1.15)
Quintile 4	63/160	0.78	(0.51, 1.17)	84/160	0.71	(0.50, 1.02)
Quintile 5	74/181	0.94	(0.63, 1.41)	92/181	0.83	(0.58, 1.18)
p-trend			0.89			0.61
per doubling in CRP		0.95	(0.88, 1.03)		0.93	(0.86, 0.99)
**Colon cancer, men only**						
Quintile 1	21/67	1	Reference	31/67	1	Reference
Quintile 2	24/69	1.26	(0.64, 2.48)	33/69	1.02	(0.59, 1.77)
Quintile 3	29/77	0.92	(0.48, 1.76)	40/77	0.69	(0.40, 1.19)
Quintile 4	27/71	0.98	(0.50, 1.93)	39/71	0.79	(0.45, 1.37)
Quintile 5	30/77	1.00	(0.51, 1.96)	40/77	0.74	(0.42, 1.30)
p-trend			0.80			0.35
per doubling in CRP		0.98	(0.86, 1.11)		0.92	(0.82, 1.02)
**Colon cancer, women only**						
Quintile 1	36/85	1	Reference	40/85	1	Reference
Quintile 2	21/70	0.62	(0.33, 1.18)	26/70	0.65	(0.37, 1.15)
Quintile 3	25/71	0.83	(0.44, 1.56)	35/71	0.99	(0.57, 1.70)
Quintile 4	36/89	0.60	(0.33, 1.09)	45/89	0.61	(0.36, 1.04)
Quintile 5	44/104	0.69	(0.38, 1.24)	52/104	0.80	(0.48, 1.35)
p-trend			0.48			0.74
per doubling in CRP		0.88	(0.78, 0.99)		0.91	(0.82, 1.00)
p-interaction by sex in colon cancer			0.39			0.60
**Rectal cancer**						
Quintile 1	34/96	1	Reference	41/96	1	Reference
Quintile 2	44/106	1.05	(0.62, 1.76)	55/106	0.98	(0.62, 1.56)
Quintile 3	33/101	0.77	(0.45, 1.32)	41/101	0.78	(0.48, 1.26)
Quintile 4	31/87	0.89	(0.50, 1.58)	40/87	0.94	(0.57, 1.54)
Quintile 5	20/65	0.86	(0.44, 1.70)	32/65	1.18	(0.68, 2.07)
p-trend			0.59			0.45
per doubling in CRP		0.93	(0.83, 1.05)		1.01	(0.91, 1.12)
p-interaction by location			0.61			0.41
**Rectal cancer, men only**						
Quintile 1	22/50	1	Reference	22/50	1	Reference
Quintile 2	32/65	1.18	(0.57, 2.43)	32/65	1.16	(0.62, 2.16)
Quintile 3	27/59	1.06	(0.49, 2.27)	27/59	0.93	(0.47, 1.81)
Quintile 4	24/43	1.02	(0.44, 2.36)	24/43	1.34	(0.66, 2.75)
Quintile 5	18/31	1.19	(0.46, 3.05)	18/31	1.51	(0.70, 3.27)
p-trend			0.87			0.25
per doubling in CRP		0.97	(0.82, 1.15)		1.06	(0.92, 1.22)
p-interaction by location in men			0.59			0.06
**Rectal cancer, women only**						
Quintile 1	16/46	1	Reference	19/46	1	Reference
Quintile 2	20/41	1.05	(0.42, 2.63)	23/41	1.05	(0.48, 2.32)
Quintile 3	10/42	0.40	(0.14, 1.11)	14/42	0.47	(0.19, 1.15)
Quintile 4	13/44	0.60	(0.23, 1.57)	16/44	0.62	(0.27, 1.42)
Quintile 5	8/34	0.41	(0.13, 1.30)	14/34	0.75	(0.29, 1.95)
p-trend			0.12			0.47
per doubling in CRP		0.85	(0.69, 1.03)		0.90	(0.76, 1.07)
p-interaction by location in women			0.41			0.64
p-interaction by sex in rectal cancer			0.13			0.27
**Stage I or II**						
Quintile 1	33/131	1	Reference	38/131	1	Reference
Quintile 2	16/107	0.62	(0.33, 1.18)	25/107	0.73	(0.43, 1.24)
Quintile 3	23/115	0.70	(0.39, 1.25)	33/115	0.75	(0.45, 1.24)
Quintile 4	14/100	0.58	(0.29, 1.16)	26/100	0.72	(0.42, 1.26)
Quintile 5	25/111	0.82	(0.44, 1.55)	42/111	0.98	(0.58, 1.67)
p-trend			0.97			0.52
per doubling in CRP		0.92	(0.82, 1.04)		0.97	(0.87, 1.07)
**Stage III or IV**						
Quintile 1	52/81	1	Reference	60/81	1	Reference
Quintile 2	60/99	0.76	(0.51, 1.15)	71/99	0.76	(0.52, 1.10)
Quintile 3	57/103	0.72	(0.47, 1.09)	71/103	0.70	(0.48, 1.03)
Quintile 4	67/119	0.73	(0.49, 1.09)	81/119	0.68	(0.47, 0.99)
Quintile 5	62/113	0.72	(0.47, 1.11)	72/113	0.71	(0.48, 1.05)
p-trend			0.36			0.27
per doubling in CRP		0.93	(0.86, 1.01)		0.93	(0.86, 1.00)
p-interaction by stage			0.91			0.19

Stratified by country with time since colorectal cancer diagnosis as underlying time variable and adjusted for age at diagnosis (in years as a continuous variable), sex, smoking status (never, former, current, unknown), body mass index (kg/m^2^) and physical activity (MET-hours/week) tumor stage (I-IV, unknown), grade of tumor differentiation (well differentiated, moderately differentiated, poorly differentiated, or unknown), location of primary tumor (colon or rectum), and year of diagnosis; (stratification variable omitted from model)

We also did not find an association when we used cut-offs of CRP originally established for cardiovascular disease prediction (< 1.0, 1.0-<3.0, or ≥ 3.0 mg/dL, Supplemental Table 1). Although the lowest HRs were observed in the 3rd quintile and in the middle category of established CRP cut-offs (1.0-<3.0 mg/L), there was no indication for non-linearity when adding quadratic or cubic terms to the model (data not shown). In sensitivity analyses (Supplemental Table 2), results were similar after excluding cases who were diagnosed with CRC during the first year (*n* = 123 excluded; highest versus lowest quintile, CRC mortality: HR 0.85, 95% CI 0.60, 1.21; all-cause mortality HR 0.83, 95% 0.61, 1.13) or first and second years after recruitment (*n* = 268 excluded; CRC mortality: HR 0.97, 95% CI 0.67, 1.41; all-cause mortality HR 0.91, 95% CI 0.65, 1.27). In analyses restricted to participants who were diagnosed with CRC within the first year after recruitment (*n* = 123), higher CRP was significantly associated with CRC (HR 1.77, 95% CI 1.19, 2.63) and overall mortality (1.42, 95% CI 1.09, 1.86). Analyses restricted to participants diagnosed within the first two years after recruitment (*n* = 268), no association was observed for either CRC (HR 0.91, 95% CI 0.78, 1.08) or overall mortality (0.94, 95% CI 0.81, 1.08). Associations were not altered after excluding participants with CRP concentrations ≥ 10 mg/L (*n* = 99 excluded; CRC mortality HR 1.01, 95% CI 0.69, 1.46; all-cause mortality HR 0.94, 95% CI 0.67, 1.30), diabetics (*n* = 123 excluded; CRC mortality HR 1.01, 95% CI 0.71, 1.42; all-cause mortality HR 0.94, 95% CI 0.69,1.28) or participants with family history of CRC (*n* = 27 excluded; CRC mortality HR 0.93, 95% CI 0.67, 1.29; all-cause mortality HR 0.92, 95% CI 0.69, 1.24).

Similarly, as for the main analysis, pre-diagnostic CRP was not significantly associated with risk of cancer-specific or all-cause mortality when stratified by sex or tumor location (Table 2). Also, no statistically significant interactions by sex or location were observed. When stratified by tumor stage, pre-diagnostic CRP was also not significantly associated with CRC mortality or all-cause mortality in persons with either tumor stage I/II or tumor stage III/IV, and no statistically significant interactions by tumor stage were observed (all p-interaction ≥ 0.19, Table 2). There was no evidence of interaction by BMI or waist circumference (all p-interaction ≥ 0.57, Supplemental Table 3) and also not by red or processed meat intake (all p-interaction ≥ 0.07).

**Fig. 1:**
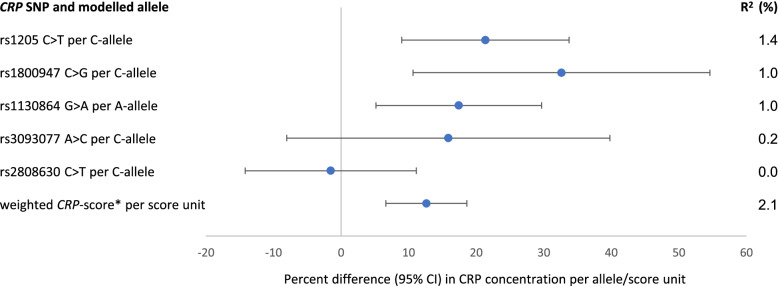
Association between CRP genetic variation and CRP concentrations in *n* = 822 individuals with CRP and CRP SNP information. * Based on SNPs rs1205, rs180047, rs1130864 and rs3093077 with estimated coefficients as weights

In the present population of individuals with CRC, the weighted *CRP*-score was associated with 13% (95% CI 7%, 19%) higher circulating CRP levels and explained 2.1% of inter-individual variation in CRP concentrations (Fig. 1). The C-allele of *CRP* SNP rs1205 was associated with 21% (95% CI 9%, 34%) higher CRP levels and explained 1.4% of inter-individual variation in CRP concentrations. The *CRP*-score was not significantly associated with CRC mortality (HR per score unit 0.95, 95% CI 0.86, 1.05) or all-cause mortality (HR 0.98, 95% CI 0.90, 1.07, Table 3). The individual *CRP* SNPs associated with circulating CRP were not associated with CRC or all-cause mortality, except that for SNP rs1205, significant inverse associations were observed with CRC mortality (comparing the CT and CC genotypes with TT genotype, HR 0.54, 95% CI 0.35, 0.83 and HR 0.58, 95% CI 0.38, 0.88, respectively) and all-cause mortality (HR 0.58, 95% CI 0.40, 0.85 and 0.64, 95% CI 0.44, 0.92, respectively). When the genotypes of SNP rs1205 were coded comparing the TT genotype with the CT and CC genotypes combined - analogous to the two previous publications [19, 20] on *CRP* genotypes and CRC mortality - significant positive associations were observed with CRC mortality (HR 1.79, 95% CI 1.20, 2.67) as well as all-cause mortality (HR 1.64, 95% CI 1.15, 2.34). The associations observed for *CRP* genetic variation did not change substantially after stratification by sex, tumor location or tumor stage (Supplemental Tables 4, 5 and 6).

**Table 3:** Association between genetic predisposition to higher circulating C-reactive protein (CRP) and colorectal cancer outcome

	CRC mortality			All-cause mortality		
	N event/N total	HR	(95% CI)	N event/N total	HR	(95% CI)
**Weighted** ***CRP*-score**						
Tertile 1	99/271	1	Reference	124/271	1	Reference
Tertile 2	72/256	0.70	(0.50, 0.96)	97/256	0.68	(0.51, 0.90)
Tertile 3	94/289	0.86	(0.63, 1.17)	125/289	0.92	(0.70, 1.19)
p-trend			0.44			0.33
per score unit		0.95	(0.86, 1.05)		0.98	(0.90, 1.07)
**rs1205**						
TT	35/82	1	Reference	41/82	1	Reference
CT	109/347	0.54	(0.35, 0.83)	144/347	0.58	(0.40, 0.85)
CC	121/387	0.58	(0.38, 0.88)	161/387	0.64	(0.44, 0.92)
p-trend			0.12			0.21
per C allele		0.85	(0.70, 1.04)	0.89	0.89	(0.75, 1.07)
TT vs. CT + CC (analogous to previous publications)		1.79	(1.20, 2.67)		1.64	(1.15, 2.34)
**rs1800947**						
CG/GG	35/108	1	Reference	43/108	1	Reference
CC	230/707	0.99	(0.67, 1.46)	303/707	1.06	(0.75, 1.49)
p-trend			0.94			0.58
per C allele		1.01	(0.71, 1.46)		1.09	(0.80, 1.50)
**rs1130864**						
GG	122/362	1	Reference	156/362	1.00	Reference
GA	115/363	0.88	(0.67, 1.16)	148/363	0.89	(0.70, 1.14)
AA	28/91	0.98	(0.63, 1.53)	42/91	1.17	(0.81, 1.68)
p-trend			0.61			0.85
per A allele		0.95	(0.78, 1.16)		1.02	(0.86, 1.21)
**rs3093077**						
AA	236/715	1	Reference	308/715	1	Reference
AC/CC	29/101	0.84	(0.56, 1.26)	38/101	0.80	(0.56, 1.14)
p-trend			0.45			0.24
per C allele		0.86	(0.58, 1.27)		0.81	(0.58, 1.15)

Stratified by country with time since colorectal cancer diagnosis as underlying time variable and adjusted for age at diagnosis (in years as a continuous variable), sex, tumor stage (I-IV, unknown), grade of tumor differentiation (well differentiated, moderately differentiated, poorly differentiated, or unknown), location of primary tumor (colon or rectum), and year of diagnosis

Genotypes associated with lower CRP-concentrations are used as reference category unless indicated otherwise

## Discussion

In this prospective study, pre-diagnostic CRP concentrations were not associated with risk of CRC-specific or all-cause mortality in participants diagnosed with CRC, colon or rectal cancer. *CRP* genetic predisposition to higher circulating CRP concentrations as reflected by the weighted *CRP*-score was not significantly associated with CRC-specific mortality. The CT and CC genotypes (compared with TT genotype) of *CRP* SNP rs1205 were significantly inversely associated with CRC-specific and all-cause mortality, while no associations were observed with the other CRP-associated *CRP* tagging SNPs.

Our finding that pre-diagnostic CRP concentrations were not associated with mortality in persons with CRC is in line with those reported by two previous studies [19, 20]. In the Copenhagen City Heart study, pre-diagnostic CRP concentrations were not significantly associated with risk of early death in persons with CRC, but these observations were based on a small number of persons (*n* = 173) [18]. Another study on CRP and CRC survival using data from the large Apolipoprotein Mortality Risk Study (AMORIS) from the greater Stockholm area was based on a larger group of persons with CRC (*n* = 4764), in whom baseline CRP concentrations were not related to CRC or all cause death [17]. However, in this large study, CRP was not measured using a high sensitivity assay, meaning that potentially informative subclinical CRP concentrations were not investigated. Given the current evidence including the results of the present analysis, pre-diagnostic CRP concentrations therefore do not seem to play a major role in survival in individuals with CRC.

While most *CRP* genetic variants investigated in our study were not associated with CRC-specific or all-cause mortality in individuals with CRC, we observed significant inverse associations for carrying a C-allele of rs1205 and no significant associations for the *CRP* genetic score associated with higher CRP. In the recent large Mendelian Randomization analysis within the International Survival Analysis in Colorectal Cancer Consortium (ISACC), a one unit increase in the genetic risk score based on 52 CRP-associated genome-wide variants was non-significantly associated with fewer deaths due to CRC, which is in line with our observation for the CRP genetic score and CRC-specific mortality [21]. In terms of rs1205, we observed that the TT genotype (the least frequent genotype, previously associated with the lowest CRP concentrations) compared with the TC and CC genotypes was associated with a higher risk of CRC-specific and all-cause mortality. Two previous studies observed an association between rs1205 (G > A polymorphism) and survival in persons with CRC [19, 20]. In a study based on CRC patients from two population-based studies in the US (*n* = 1364 colon cancer and *n* = 697 rectal cancer patients) with some ethnic heterogeneity, the AA genotype (minor genotype) versus GG/GA genotypes (corresponding to TT versus CC/TC genotypes in our analysis due to genotyping on alternative DNA strand) was associated with lower cancer-specific and all-cause mortality among persons with colon but not in those with rectal cancer [19]. In another study in individuals with CRC from Taiwan, the AA genotype versus GG/GA genotypes was associated lower cancer-specific and overall survival, i.e. with higher cancer-specific and all-cause mortality [20]. Thus, while the findings on colon cancer in the US study are in the opposite direction of our findings, the results of the Asian study are in line with our observations. It should be noted, however, that allele frequencies of rs1205 in the Taiwanese study were different from ours, in the sense that the A-allele (corresponding to the T-allele in our analysis) was the major allele, whereas in our study the C-allele was the major allele. While in the Taiwanese study the association between rs1205 and circulating CRP levels could not be investigated, a Chinese study provided evidence that the C-allele of rs1205 is associated with higher CRP concentrations also in Asian populations [29]. In the Mendelian Randomization analysis in ISACC, rs2794520, a proxy SNP of rs1205, was not significantly associated with CRC mortality [21].With respect to CRC incidence, a recent meta-analysis including eight studies on rs1205 observed overall no association (pooled OR TT + TC vs. CC 1.01, 95% CI 0.94, 1.10) with strong indication for heterogeneity (p-heterogeneity 0.003) [30].

As previously shown in control participants from the nested case-control study on CRC in EPIC [11] and elsewhere [31], the C-allele (or corresponding G-allele) of rs1205 is associated with higher circulating CRP (EPIC data: 18% higher CRP per C-allele). Consequently, the TT (or corresponding AA) genotype, which was associated with higher mortality compared with the CC and CT genotypes combined in our study and in the study by Yang et al., has been associated with lower CRP concentrations. We also observed that the *CRP* genetic score indicating a genetic predisposition to lifelong higher CRP concentrations was non-significantly associated with lower CRC-specific mortality. These results are in contrast to the overall hypothesis that inflammatory processes affect not only carcinogenesis but also cancer progression, for instance through angiogenesis due to induction of vascular endothelial growth factor [32]. To provide possible explanation of these contrasting findings, further research – for example into progression-related tumor behavior in individuals with CRC with different genetic predisposition to inflammatory response - is required. On the one hand, genetic susceptibility to higher inflammation as reflected by higher lifelong CRP concentrations may have an effect on prognosis in persons with CRC. On the other hand, pleiotropic effects of rs1205 may have also played a role. For example, it has been observed that rs1205 is associated with two-hour glucose post oral glucose tolerance test, which might affect survival in persons with CRC in CRP-independent ways [33], as there is evidence suggesting that insulin resistance increases progression and worsens prognosis in several types of cancer, including CRC [34].

Strengths of our study include the prospective study design and the availability of measured pre-diagnostic high-sensitivity CRP and tagging SNPs in the *CRP* gene in one sample, which enabled us to estimate the association between *CRP* SNPs and CRP-concentrations directly. Despite a relatively large number of persons with CRC, the sample size was limited for genetic and subgroup analyses. Given our calculation of statistical power, we had sufficient power (0.8) to detect a HR of 1.08 or 0.93 for the analyses of circulating CRP and CRC mortality, but for genetic analyses, only a HR of 1.14 or 0.88 could be detected with sufficient power. Further limitations of our study include that information on treatment of CRC, which may impact CRC mortality, was not available, although this may be partly accounted for by adjustment for tumor stage and grade of tumor differentiation as well as by stratification by country since treatment strategies may differ in European countries. There was a certain proportion of missing information regarding tumor stage and grade, which were coded as separate category during analysis. In a previous analysis in the same dataset it was shown that various approaches to account for these uncertainties including complete case analysis and multiple imputation demonstrated robustness of associations [35]. It is a further limitation that information on regular intake of anti-inflammatory drugs was not available. In terms of circulating CRP, we cannot exclude that non-differential misclassification may have occurred in our analysis, since CRP was measured only at one time point pre-diagnostically, and this measurement may also have been affected by acute inflammatory response due to infection. This potential misclassification was circumvented by using genetic variants associated with circulating CRP. However, in order to investigate whether genetic predisposition to lifelong higher CRP concentrations plays a role in mortality in persons with CRC, our study was limited in the sense that only genetic variants in the *CRP* gene could be included in the *CRP*-score, although there is evidence from genome-wide association studies that also SNPs in other loci, including those implicating pathways related to metabolic syndrome and immune system, are associated with circulating CRP [36]. Our primary outcome was mortality from CRC and competing risks due to death from other causes were present. However, our study aimed at elucidating an etiologic question, in which case it has been suggested that cause-specific hazards regression, as we applied here, is the appropriate statistical approach, even in the presence of competing risks [37]. We can also not exclude that collider stratification bias impaired our ability to detect a potential positive association between circulating CRP and survival in persons with CRC. This would be the case if an unmeasured confounder associated with both CRC incidence and post-diagnosis mortality led to differential distribution of such confounder in our selected study collective of CRC patients compared with the general population [38–40]. In terms of smoking – a measured confounder – there was some indication for differential distribution, since we observed decreasing proportion of smokers across CRP quintiles, which is in contrast to the previous analysis on CRC incidence in EPIC where increasing proportion of smokers was observed across CRP quintiles in control participants and overall proportion of smokers was lower in controls than in CRC cases [10]. In our analyses, we controlled for potential confounding by smoking in all models.

In conclusion, this study, which poses the so far largest analysis of circulating pre-diagnostic high-sensitivity CRP in individuals with CRC, does not indicate an association of pre-diagnostic CRP concentrations with CRC-specific or all-cause mortality. While no association between most CRP-associated *CRP* genetic variants and CRC-specific or all-cause mortality was observed, we found some evidence for associations with rs1205 as well as with a *CRP*-score associated with higher circulating CRP and lower mortality, which deserve further scientific attention.

## Supplementary Information

**Additional file 1.**

## Data Availability

The data that support the findings of this study are available from International Institute for Research on Cancer (IARC), but restrictions apply to the availability of these data, which were used under license for the current study, and so are not publicly available. Data are however available from the authors upon reasonable request and with permission of IARC. For information on how to submit an application for gaining access to EPIC data and/or bio-specimens, please follow the instructions at http://epic.iarc.fr/access/index.php.
