# Supplementary material for: Pre-diagnostic C-reactive protein concentrations, CRP genetic variation and mortality among individuals with colorectal cancer in Western European populations
Source: BMC Cancer. 2022 Jun 24;22:695. doi: 10.1186/s12885-022-09778-9 (PMC9229883; doi:10.1186/s12885-022-09778-9)
Supplement: Supplementary file 1 — Additional file 1. [file 12885_2022_9778_MOESM1_ESM.docx]

Supplemental table 1. Association between pre-diagnostic C-reactive protein (CRP) categories and colorectal cancer outcome

|  | CRC mortality | | | All-cause mortality | | |
| --- | --- | --- | --- | --- | --- | --- |
|  | N event/N Total | HR | (95% CI) | N event/N Total | HR | (95% CI) |
| **Colorectal cancer** | |  |  |  |  |  |
| <1 mg/L | 121/324 | 1 | Reference | 148/324 | 1 | Reference |
| 1-3 mg/L | 129/376 | 0.77 | (0.59, 1.01) | 172/376 | 0.78 | (0.61, 0.98) |
| >3 mg/L | 205/535 | 0.84 | (0.65, 1.08) | 270/535 | 0.84 | (0.67, 1.05) |
| p-trend |  |  | 0.45 |  |  | 0.42 |
| **Colorectal cancer, men only** | | |  |  |  |  |
| <1 mg/L | 51/153 | 1 | Reference | 69/153 | 1.00 | Reference |
| 1-3 mg/L | 79/211 | 0.86 | (0.58, 1.26) | 103/211 | 0.78 | (0.56, 1.09) |
| >3 mg/L | 96/245 | 0.96 | (0.65, 1.41) | 134/245 | 0.9 | (0.65, 1.25) |
| p-trend |  |  | 0.89 |  |  | 1.00 |
| **Colorectal cancer, women only** | | |  |  |  |  |
| <1 mg/L | 70/171 | 1 | Reference | 79/171 | 1.00 | Reference |
| 1-3 mg/L | 50/165 | 0.65 | (0.43, 0.98) | 69/165 | 0.77 | (0.54, 1.10) |
| >3 mg/L | 109/290 | 0.66 | (0.46, 0.97) | 136/290 | 0.74 | (0.52, 1.03) |
| p-trend |  |  | 0.0905 |  |  | 0.1381 |
| p-interaction by sex | |  | 0.35 |  |  | 0.16 |
| **Colon cancer** | |  |  |  |  |  |
| <1 mg/L | 70/191 | 1 | Reference | 88/191 | 1.00 | Reference |
| 1-3 mg/L | 74/221 | 0.8 | (0.56, 1.15) | 101/221 | 0.76 | (0.56, 1.05) |
| >3 mg/L | 149/368 | 0.87 | (0.62, 1.21) | 192/368 | 0.80 | (0.59, 1.07) |
| p-trend |  |  | 0.67 |  |  | 0.33 |
| **Colon cancer, men only** | |  |  |  |  |  |
| <1 mg/L | 25/82 | 1 | Reference | 37/82 | 1.00 | Reference |
| 1-3 mg/L | 44/117 | 1.03 | (0.58, 1.82) | 59/117 | 0.79 | (0.49, 1.27) |
| >3 mg/L | 62/162 | 0.95 | (0.54, 1.68) | 87/162 | 0.76 | (0.48, 1.21) |
| p-trend |  |  | 0.78 |  |  | 0.38 |
| **Colon cancer, women only** | | |  |  |  |  |
| <1 mg/L | 45/109 | 1 | Reference | 51/109 | 1.00 | Reference |
| 1-3 mg/L | 30/104 | 0.56 | (0.33, 0.96) | 42/104 | 0.68 | (0.43, 1.08) |
| >3 mg/L | 87/206 | 0.65 | (0.40, 1.05) | 105/206 | 0.71 | (0.46, 1.09) |
| p-trend |  |  | 0.2371 |  |  | 0.2502 |
| p-interaction by sex in colon cancer | | | 0.98 |  |  | 0.67 |
| **Rectal cancer** | |  |  |  |  |  |
| <1 mg/L | 51/133 | 1 | Reference | 60/133 | 1.00 | Reference |
| 1-3 mg/L | 55/155 | 0.78 | (0.50, 1.20) | 71/155 | 0.84 | (0.57, 1.24) |
| >3 mg/L | 56/167 | 0.74 | (0.46, 1.18) | 78/167 | 0.89 | (0.60, 1.34) |
| p-trend |  |  | 0.27 |  |  | 0.74 |
| p-interaction by location | |  | 0.22 |  |  | 0.88 |
| **Rectal cancer, men only** | |  |  |  |  |  |
| <1 mg/L | 26/71 | 1 | Reference | 32/71 | 1.00 | Reference |
| 1-3 mg/L | 35/94 | 0.91 | (0.51, 1.65) | 44/94 | 0.9 | (0.53, 1.50) |
| >3 mg/L | 34/83 | 0.85 | (0.44, 1.67) | 47/83 | 1.13 | (0.64, 2.01) |
| p-trend |  |  | 0.66 |  |  | 0.54 |
| p-interaction by location in men | | | 0.46 |  |  | 0.13 |
| **Rectal cancer, women only** | | |  |  |  |  |
| <1 mg/L | 25/62 | 1 | Reference | 28/62 | 1.00 | Reference |
| 1-3 mg/L | 20/61 | 0.67 | (0.31, 1.47) | 27/61 | 0.67 | (0.33, 1.34) |
| >3 mg/L | 22/84 | 0.47 | (0.21, 1.03) | 31/84 | 0.51 | (0.25, 1.00) |
| p-trend |  |  | 0.07 |  |  | 0.07 |
| p-interaction by sex in rectal cancer | | | 0.09 |  |  | 0.11 |
| p-interaction by location in women | | | 0.10 |  |  | 0.11 |
| **Stage I or II** |  |  |  |  |  |  |
| <1 mg/L | 38/162 | 1 | Reference | 47/162 | 1.00 | Reference |
| 1-3 mg/L | 28/169 | 0.62 | (0.36, 1.04) | 39/169 | 0.61 | (0.38, 0.95) |
| >3 mg/L | 45/233 | 0.76 | (0.45, 1.29) | 78/233 | 0.88 | (0.57, 1.35) |
| p-trend |  |  | 0.60 |  |  | 0.81 |
| **Strage III or IV** | |  |  |  |  |  |
| <1 mg/L | 73/112 | 1 | Reference | 82/112 | 1.00 | Reference |
| 1-3 mg/L | 86/157 | 0.67 | (0.48, 0.95) | 109/157 | 0.71 | (0.52, 0.98) |
| >3 mg/L | 139/246 | 0.74 | (0.53, 1.02) | 164/246 | 0.73 | (0.54, 0.99) |
| p-trend |  |  | 0.27 |  |  | 0.16 |
| p-interaction by stage | |  | 0.96 |  |  | 0.16 |

Adjusted for age at diagnosis (in years as a continuous variable), sex, smoking status, body mass index (kg/m^2^), physical activity (MET-hours/week), tumor stage (I-IV, unknown), grade of tumor differentiation (well differentiated, moderately differentiated, poorly differentiated, or unknown), location of primary tumor (colon or rectum), and year of diagnosis; (stratification variable omitted from model)

Supplemental table 2a Association between pre-diagnostic C-reactive protein (CRP) concentrations and colorectal cancer outcome, with different time length between recruitment (blood collection) and CRC diagnosis

|  | CRC mortality | | | All-cause mortality | | |
| --- | --- | --- | --- | --- | --- | --- |
|  | N event/N Total | OR | (95% CI) | N event/N Total | OR | (95% CI) |
| **Cases diagnosed with CRC within the first year after recruitment excluded (n=1112)** | | | | | | |
| Quintile 1 | 89/232 | 1 | Reference | 107/232 | 1 | Reference |
| Quintile 2 | 82/230 | 0.95 | (0.68, 1.31) | 106/230 | 0.93 | (0.69, 1.24) |
| Quintile 3 | 79/223 | 0.78 | (0.56, 1.08) | 105/223 | 0.78 | (0.58, 1.05) |
| Quintile 4 | 82/221 | 0.76 | (0.54, 1.06) | 110/221 | 0.78 | (0.58, 1.05) |
| Quintile 5 | 77/206 | 0.85 | (0.60, 1.21) | 100/206 | 0.83 | (0.61, 1.13) |
| p-trend |  |  | 0.45 |  |  | 0.32 |
| per doubling in CRP | | 0.93 | (0.87, 0.99) |  | 0.93 | (0.88, 0.99) |
| **Analyses restricted to cases diagnosed with CRC within the first year after recruitment (n=123)** | | | | | | |
| per doubling in CRP** | | 1.77 | (1.19, 2.63) |  | 1.42 | (1.09, 1.86) |
|  |  |  |  |  |  |  |
| **Cases diagnosed with CRC within the first 2 years after recruitment excluded (n=967)** | | | | | | |
| Quintile 1 | 81/215 | 1 | Reference | 98/215 | 1 | Reference |
| Quintile 2 | 63/196 | 0.99 | (0.69, 1.41) | 83/196 | 0.95 | (0.69, 1.30) |
| Quintile 3 | 68/194 | 0.85 | (0.60, 1.22) | 93/194 | 0.86 | (0.63, 1.18) |
| Quintile 4 | 73/189 | 0.85 | (0.59, 1.22) | 99/189 | 0.85 | (0.62, 1.16) |
| Quintile 5 | 66/173 | 0.97 | (0.67, 1.41) | 85/173 | 0.91 | (0.65, 1.27) |
| p-trend |  |  | 0.93 |  |  | 0.66 |
| per doubling in CRP | | 0.95 | (0.89, 1.02) |  | 0.96 | (0.90, 1.02) |
| **Analyses restricted to cases diagnosed with CRC within the first 2 years after recruitment (n=268)** | | | | | | |
| per doubling in CRP** | | 0.91 | (0.78, 1.08) |  | 0.94 | (0.81, 1.08) |

** no quintile analyses performed due to low number of cases included in the restricted analyses

Supplemental table 2b Association between pre-diagnostic C-reactive protein (CRP) concentrations and colorectal cancer outcome, with high CRP (≥10 mg/l, n=99) excluded

|  | CRC mortality | | | All-cause mortality | | |
| --- | --- | --- | --- | --- | --- | --- |
|  | N event/N Total | OR | (95% CI) | N event/N Total | OR | (95% CI) |
| **CRP≥10 mg/L excluded (n=1136)** | | |  |  |  |  |
| Quintile 1 | 91/248 | 1 | Reference | 112/248 | 1 | Reference |
| Quintile 2 | 89/245 | 0.98 | (0.72, 1.35) | 114/245 | 0.95 | (0.72, 1.25) |
| Quintile 3 | 87/249 | 0.81 | (0.59, 1.11) | 116/249 | 0.80 | (0.60, 1.06) |
| Quintile 4 | 94/247 | 0.86 | (0.62, 1.18) | 124/247 | 0.85 | (0.64, 1.13) |
| Quintile 5 | 57/147 | 1.01 | (0.69, 1.46) | 72/147 | 0.94 | (0.67, 1.30) |
| p-trend |  |  | 0.99 |  |  | 0.75 |
| per doubling in CRP | | 0.95 | (0.88, 1.02) |  | 0.94 | (0.88, 1.00) |

Supplemental table 2c Association between prediagnostic CRP concentrations and colorectal cancer outcome, diabetics (n=123) excluded

|  | CRC mortality | | | All-cause mortality | | |
| --- | --- | --- | --- | --- | --- | --- |
|  | N event/N Total | OR | (95% CI) | N event/N Total | OR | (95% CI) |
| **Diabetics excluded (n=1112)** | | |  |  |  |  |
| Quintile 1 | 83/236 | 1 | Reference | 103/236 | 1 | Reference |
| Quintile 2 | 83/231 | 1.09 | (0.78, 1.52) | 107/231 | 1.03 | (0.77, 1.38) |
| Quintile 3 | 81/232 | 0.84 | (0.60, 1.17) | 107/232 | 0.8 | (0.60, 1.07) |
| Quintile 4 | 83/211 | 0.91 | (0.65, 1.28) | 105/211 | 0.86 | (0.64, 1.16) |
| Quintile 5 | 80/202 | 1.01 | (0.71, 1.42) | 99/202 | 0.94 | (0.69, 1.28) |
| p-trend |  |  | 0.97 |  |  | 0.71 |
| per doubling in CRP | | 0.96 | (0.90, 1.03) |  | 0.95 | (0.90, 1.01) |

Supplemental table 2d Association between prediagnostic CRP concentrations and colorectal cancer outcome, participants with family history of CRC (n=27) excluded

|  | CRC mortality | | | All-cause mortality | | |
| --- | --- | --- | --- | --- | --- | --- |
|  | N event/N Total | OR | (95% CI) | N event/N Total | OR | (95% CI) |
| **Participants with family history of CRC excluded (n=1208)** | | | | |  |  |
| Quintile 1 | 90/242 | 1 | Reference | 110/242 | 1 | Reference |
| Quintile 2 | 87/241 | 0.96 | (0.70, 1.32) | 112/241 | 0.94 | (0.71, 1.24) |
| Quintile 3 | 83/245 | 0.77 | (0.56, 1.06) | 112/245 | 0.78 | (0.59, 1.03) |
| Quintile 4 | 90/241 | 0.84 | (0.61, 1.16) | 118/241 | 0.83 | (0.63, 1.10) |
| Quintile 5 | 92/239 | 0.93 | (0.67, 1.29) | 121/239 | 0.92 | (0.69, 1.24) |
| p-trend |  |  | 0.86 |  |  | 0.89 |
| per doubling in CRP | | 0.95 | (0.89, 1.01) |  | 0.96 | (0.91, 1.01 |

Adjusted for age at diagnosis (in years as a continuous variable), sex, smoking status, body mass index (kg/m^2^), physical activity (MET-hours/week), tumor stage (I-IV, unknown), grade of tumor differentiation (well differentiated, moderately differentiated, poorly differentiated, or unknown), location of primary tumor (colon or rectum), and year of diagnosis; (stratification variable omitted from model)

Supplemental table 3a. Association between pre-diagnostic CRP concentrations and colorectal cancer outcome, stratified by BMI categories

|  | CRC mortality | | | All-cause mortality | | |
| --- | --- | --- | --- | --- | --- | --- |
|  | N event/N Total | OR | (95% CI) | N event/N Total | OR | (95% CI) |
| **BMI <25 kg/m^2^** |  |  |  |  |  |  |
| Quintile 1 | 46/146 | 1 | Reference | 59/146 | 1 | Reference |
| Quintile 2 | 43/108 | 1.4 | (0.84, 2.33) | 50/108 | 1.12 | (0.71, 1.75) |
| Quintile 3 | 26/86 | 0.89 | (0.51, 1.56) | 36/86 | 0.86 | (0.54, 1.39) |
| Quintile 4 | 30/77 | 0.95 | (0.55, 1.65) | 40/77 | 0.91 | (0.57, 1.46) |
| Quintile 5 | 20/50 | 1.17 | (0.63, 2.16) | 24/50 | 1.05 | (0.62, 1.80) |
| p-trend |  |  | 0.92 |  |  | 0.89 |
| per doubling in CRP | | 0.97 | (0.87, 1.07) |  | 0.97 | (0.88, 1.06) |
| **BMI 25-<30 kg/m^2^** | |  |  |  |  |  |
| Quintile 1 | 38/87 | 1 | Reference | 45/87 | 1 | Reference |
| Quintile 2 | 34/108 | 0.67 | (0.40, 1.13) | 45/108 | 0.76 | (0.48, 1.20) |
| Quintile 3 | 46/122 | 0.63 | (0.38, 1.03) | 58/122 | 0.66 | (0.42, 1.02) |
| Quintile 4 | 40/103 | 0.81 | (0.49, 1.34) | 49/103 | 0.83 | (0.53, 1.31) |
| Quintile 5 | 43/119 | 0.68 | (0.41, 1.14) | 57/119 | 0.79 | (0.50, 1.24) |
| p-trend |  |  | 0.60 |  |  | 0.90 |
| per doubling in CRP | | 0.93 | (0.85, 1.03) |  | 0.96 | (0.88, 1.04) |
| **BMI ≥30 kg/m^2^** | |  |  |  |  |  |
| Quintile 1 | 7/15 | 1 | Reference | 8/15 | 1 | Reference |
| Quintile 2 | 12/29 | 1.21 | (0.38, 3.84) | 19/29 | 1.48 | (0.54, 4.03) |
| Quintile 3 | 15/41 | 1.32 | (0.43, 4.08) | 22/41 | 1.55 | (0.57, 4.16) |
| Quintile 4 | 24/67 | 1.05 | (0.35, 3.13) | 35/67 | 1.24 | (0.47, 3.24) |
| Quintile 5 | 31/77 | 1.61 | (0.55, 4.73) | 43/77 | 1.68 | (0.64, 4.40) |
| p-trend |  |  | 0.34 |  |  | 0.43 |
| per doubling in CRP | | 1.04 | (0.86, 1.27) |  | 1.02 | (0.87, 1.20) |
| p-interaction by BMI | |  | 0.93 |  |  | 0.67 |

Supplemental table 3b. Association between prediagnostic CRP concentrations and colorectal cancer outcome, stratified by waist circumference categories

|  | CRC mortality | | | All-cause mortality | | |
| --- | --- | --- | --- | --- | --- | --- |
|  | N event/N Total | OR | (95% CI) | N event/N Total | OR | (95% CI) |
| **Waist circumference <88 cm in women, <102 cm in men** | | | | |  |  |
| Quintile 1 | 66/190 | 1 | Reference | 84/190 | 1 | Reference |
| Quintile 2 | 66/185 | 0.99 | (0.68, 1.44) | 82/185 | 0.92 | (0.66, 1.29) |
| Quintile 3 | 53/163 | 0.82 | (0.55, 1.21) | 73/163 | 0.79 | (0.56, 1.11) |
| Quintile 4 | 55/141 | 0.89 | (0.60, 1.32) | 74/141 | 0.88 | (0.63, 1.23) |
| Quintile 5 | 40/112 | 0.95 | (0.62, 1.47) | 52/112 | 0.92 | (0.63, 1.34) |
| p-trend |  |  | 0.82 |  |  | 0.78 |
| per doubling in CRP | | 0.96 | (0.89, 1.04) |  | 0.96 | (0.90, 1.03) |
|  |  |  |  |  |  |  |
| **Waist circumference ≥88 cm in women, ≥102 cm in men** | | | | |  |  |
| Quintile 1 | 25/58 | 1 | Reference | 28/58 | 1 | Reference |
| Quintile 2 | 23/60 | 0.87 | (0.45, 1.68) | 32/60 | 0.87 | (0.48, 1.56) |
| Quintile 3 | 34/86 | 0.63 | (0.34, 1.18) | 43/86 | 0.69 | (0.39, 1.20) |
| Quintile 4 | 39/106 | 0.62 | (0.33, 1.16) | 50/106 | 0.62 | (0.35, 1.08) |
| Quintile 5 | 54/134 | 0.66 | (0.36, 1.20) | 72/134 | 0.73 | (0.42, 1.25) |
| p-trend |  |  | 0.37 |  |  | 0.57 |
| per doubling in CRP | | 0.88 | (0.78, 0.99) |  | 0.92 | (0.83, 1.02) |
| p-interaction by waist circumference | | | 0.57 |  |  | 0.72 |

Supplemental table 3c. Association betweeen prediagnostic CRP concentrations and colorectal cancer outcome, stratified by red meat intake categories

|  | CRC mortality | | | All-cause mortality | | |
| --- | --- | --- | --- | --- | --- | --- |
|  | N event/N Total | HR | (95% CI) | N event/N Total | HR | (95% CI) |
| **Red meat intake <48.8 g/d** | | |  |  |  |  |
| Quintile 1 | 48/140 | 1 | Reference | 57/140 | 1 | Reference |
| Quintile 2 | 35/123 | 0.69 | (0.42, 1.13) | 44/123 | 0.70 | (0.45, 1.09) |
| Quintile 3 | 37/113 | 0.67 | (0.41, 1.09) | 47/113 | 0.69 | (0.45, 1.07) |
| Quintile 4 | 46/126 | 0.75 | (0.46, 1.22) | 64/126 | 0.78 | (0.51, 1.19) |
| Quintile 5 | 53/130 | 0.90 | (0.55, 1.47) | 64/130 | 0.87 | (0.56, 1.35) |
| p-trend |  |  | 0.65 |  |  | 0.75 |
| per doubling in CRP | | 0.95 | (0.86, 1.05) |  | 0.96 | (0.88, 1.05) |
|  |  |  |  |  |  |  |
| **Red meat intake ≥48.8 g/d** | | |  |  |  |  |
| Quintile 1 | 43/108 | 1 | Reference | 55/108 | 1 | Reference |
| Quintile 2 | 54/122 | 1.12 | (0.72, 1.76) | 70/122 | 1.09 | (0.74, 1.61) |
| Quintile 3 | 50/136 | 0.85 | (0.54, 1.34) | 69/136 | 0.84 | (0.57, 1.24) |
| Quintile 4 | 48/121 | 0.92 | (0.58, 1.44) | 60/121 | 0.83 | (0.56, 1.24) |
| Quintile 5 | 41/116 | 0.82 | (0.51, 1.33) | 60/116 | 0.83 | (0.55, 1.25) |
| p-trend |  |  | 0.29 |  |  | 0.24 |
| per doubling in CRP | | 0.94 | (0.86, 1.02) |  | 0.93 | (0.86, 1.00) |
| p-interaction by red meat intake | | | 0.22 |  |  | 0.07 |

Supplemental table 3d. Association between prediagnostic CRP concentrations and colorectal cancer outcome, stratified by processed meat intake categories

|  | CRC mortality | | | All-cause mortality | | |
| --- | --- | --- | --- | --- | --- | --- |
|  | N event/N Total | HR | (95% CI) | N event/N Total | HR | (95% CI) |
| **Processed meat intake <25.5 g/d** | | |  |  |  |  |
| Quintile 1 | 48/127 | 1 | Reference | 58/127 | 1 | Reference |
| Quintile 2 | 43/116 | 0.82 | (0.52, 1.30) | 56/116 | 0.84 | (0.57, 1.26) |
| Quintile 3 | 38/121 | 0.55 | (0.34, 0.89) | 52/121 | 0.59 | (0.38, 0.90) |
| Quintile 4 | 46/117 | 0.69 | (0.43, 1.09) | 59/117 | 0.66 | (0.44, 0.99) |
| Quintile 5 | 43/119 | 0.67 | (0.41, 1.11) | 59/119 | 0.77 | (0.50, 1.18) |
| p-trend |  |  | 0.28 |  |  | 0.51 |
| per doubling in CRP | | 0.91 | (0.83, 0.99) |  | 0.93 | (0.86, 1.01) |
| **Processed meat intake ≥25.5 g/d** | | |  |  |  |  |
| Quintile 1 | 43/121 | 1 | Reference | 54/121 | 1 | Reference |
| Quintile 2 | 46/129 | 1.31 | (0.81, 2.12) | 58/129 | 1.1 | (0.73, 1.68) |
| Quintile 3 | 49/128 | 1.09 | (0.68, 1.74) | 64/128 | 1.01 | (0.67, 1.52) |
| Quintile 4 | 48/130 | 1.13 | (0.70, 1.83) | 65/130 | 1.1 | (0.73, 1.65) |
| Quintile 5 | 51/127 | 1.26 | (0.78, 2.04) | 65/127 | 1.09 | (0.72, 1.66) |
| p-trend |  |  | 0.58 |  |  | 0.77 |
| per doubling in CRP | | 1.00 | (0.91, 1.09) |  | 0.99 | (0.91, 1.07) |
| p-interaction by processed meat intake |  |  | 0.55 |  |  | 0.91 |

Adjusted for age at diagnosis (in years as a continuous variable), sex, smoking status, body mass index (kg/m^2^), physical activity (MET-hours/week), tumor stage (I-IV, unknown), grade of tumor differentiation (well differentiated, moderately differentiated, poorly differentiated, or unknown), location of primary tumor (colon or rectum), and year of diagnosis

Supplemental table 4 Association between genetic predisposition to higher CRP concentrations and colorectal cancer outcome, models stratified by sex

|  | **Men** | | | | | | |  | **Women** | | | | | |
| --- | --- | --- | --- | --- | --- | --- | --- | --- | --- | --- | --- | --- | --- | --- |
|  | CRC mortality | | | | All-cause mortality | | |  | CRC mortality | | | All-cause mortality | | |
|  | N event/N Total | | HR | (95% CI) | N event/N Total | HR | (95% CI) |  | N event/N Total | HR | (95% CI) | N event/N Total | HR | (95% CI) |
| **Weighted CRP-score** | | |  |  |  |  |  |  |  |  |  |  |  |  |
| Tertile 1 | 45/127 | | 1.00 |  | 55/127 |  |  |  | 54/144 |  |  | 69/144 |  |  |
| Tertile 2 | 31/112 | | 0.70 | (0.50, 0.97) | 44/112 | 0.62 | (0.40, 0.98) |  | 41/144 | 0.85 | (0.54, 1.34) | 53/144 | 0.81 | (0.55, 1.20) |
| Tertile 3 | 38/128 | | 0.85 | (0.63, 1.15) | 57/128 | 0.82 | (0.54, 1.24) |  | 56/161 | 1.08 | (0.70, 1.67) | 68/161 | 1.03 | (0.70, 1.50) |
| p-trend |  | |  | 0.3219 |  |  | 0.2727 |  |  |  | 0.8291 |  |  | 0.9672 |
| per score unit |  | | 0.91 | (0.78, 1.07) |  | 0.97 | (0.85, 1.11) |  |  | 1.01 | (0.88, 1.17) |  | 1.02 | (0.91, 1.15) |
| **rs1205** |  | |  |  |  |  |  |  |  |  |  |  |  |  |
| TT | 16/40 | | 0.65 | (0.32, 1.29) | 19/40 |  |  |  | 19/42 |  |  | 22/42 |  |  |
| CT | 51/153 | | 0.53 | (0.26, 1.05) | 67/153 | 0.8 | (0.44, 1.44) |  | 58/194 | 0.60 | (0.34, 1.06) | 77/194 | 0.66 | (0.39, 1.12) |
| CC | 47/174 | | 0.53 | (0.26, 1.05) | 70/174 | 0.71 | (0.40, 1.27) |  | 74/213 | 0.77 | (0.44, 1.35) | 91/213 | 0.79 | (0.47, 1.32) |
| p-trend |  | |  | 0.08 |  |  | 0.25 |  |  |  | 0.94 |  |  | 0.96 |
| per C allele |  | | 0.75 | (0.55, 1.03) |  | 0.86 | (0.67, 1.11) |  |  | 1.01 | (0.78, 1.32) |  | 0.99 | (0.79, 1.26) |
| TT vs CT+CC (analogous to other publications) | | | 1.38 | (0.74, 2.58) |  | 1.33 | (0.77, 2.32) |  |  | 1.46 | (0.85, 2.49) |  | 1.37 | (0.84, 2.24) |
| **rs1800947** | |  |  |  |  |  |  |  |  |  |  |  |  |  |
| CG/GG | | 12/46 |  |  | 16/46 |  |  |  | 23/62 |  |  | 27/62 |  |  |
| CC | | 102/320 | 1.27 | (0.65, 2.46) | 140/320 | 1.31 | (0.75, 2.30) |  | 128/387 | 0.98 | (0.59, 1.63) | 163/387 | 1.02 | (0.65, 1.60) |
| p-trend | |  |  | 0.32 |  |  | 0.17 |  |  |  | 1.00 |  |  | 0.83 |
| per C allele | |  | 1.36 | (0.74, 2.50) |  | 1.42 | (0.86, 2.36) |  |  | 1 | (0.63, 1.58) |  | 1.05 | (0.69, 1.58) |
| **rs1130864** | |  |  |  |  |  |  |  |  |  |  |  |  |  |
| GG | | 52/171 |  |  | 66/171 |  |  |  | 70/191 |  |  | 90/191 |  |  |
| GA | | 49/152 | 1.13 | (0.74, 1.73) | 69/152 | 1.22 | (0.85, 1.76) |  | 66/211 | 0.79 | (0.54, 1.14) | 79/211 | 0.69 | (0.50, 0.95) |
| AA | | 13/44 | 0.93 | (0.47, 1.83) | 21/44 | 1.05 | (0.61, 1.83) |  | 15/47 | 1.01 | (0.55, 1.85) | 21/47 | 1.07 | (0.64, 1.79) |
| p-trend | |  |  | 0.92 |  |  | 0.55 |  |  |  | 0.53 |  |  | 0.35 |
| per A allele | |  | 1.01 | (0.76, 1.36) |  | 1.08 | (0.84, 1.38) |  |  | 0.91 | (0.69, 1.21) |  | 0.89 | (0.69, 1.14) |
| **rs3093077** | |  |  |  |  |  |  |  |  |  |  |  |  |  |
| AA | | 105/319 |  |  | 143/319 |  |  |  | 131/396 |  |  | 165/396 |  |  |
| AC/CC | | 9/48 | 0.48 | (0.23, 1.00) | 13/48 | 0.55 | (0.31, 1.01) |  | 20/53 | 1.26 | (0.76, 2.10) | 25/53 | 1.28 | (0.82, 2.01) |
| p-trend | |  |  | 0.05 |  |  | 0.02 |  |  |  | 0.45 |  |  | 0.33 |
| per C allele | |  | 0.48 | (0.23, 1.00) |  | 0.49 | (0.27, 0.89) |  |  | 1.2 | (0.75, 1.89) |  | 1.23 | (0.81, 1.86) |

Adjusted for age at diagnosis (in years as a continuous variable), tumor stage (I-IV, unknown), grade of tumor differentiation (well differentiated, moderately differentiated, poorly differentiated, or unknown), location of primary tumor (colon or rectum), and year of diagnosis

Supplemental table 5 Association between genetic predisposition to higher CRP concentrations and colorectal cancer outcome, models stratified by location

|  | **Colon cancer** | | | | | | |  | **Rectal cancer** | | | | | |
| --- | --- | --- | --- | --- | --- | --- | --- | --- | --- | --- | --- | --- | --- | --- |
|  | CRC mortality | | | | All-cause mortality | | |  | CRC mortality | | | All-cause mortality | | |
|  | N event/N Total | | HR | (95% CI) | N event/N Total | HR | (95% CI) |  | N event/N Total | HR | (95% CI) | N event/N Total | HR | (95% CI) |
| **Weighted CRP-score** | | |  |  |  |  |  |  |  |  |  |  |  |  |
| Tertile 1 | 67/179 | |  |  | 85/179 |  |  |  | 32/92 |  |  | 39/92 |  |  |
| Tertile 2 | 52/179 | | 0.69 | (0.46, 1.04) | 72/179 | 0.66 | (0.47, 0.93) |  | 20/77 | 1.13 | (0.59, 2.17) | 25/77 | 1.17 | (0.66, 2.08) |
| Tertile 3 | 69/197 | | 0.98 | (0.68, 1.42) | 93/197 | 0.99 | (0.72, 1.36) |  | 25/92 | 0.84 | (0.43, 1.66) | 32/92 | 0.96 | (0.53, 1.73) |
| p-trend |  | |  | 0.75 |  |  | 0.72 |  |  |  | 0.37 |  |  | 0.46 |
| per score unit |  | | 0.99 | (0.87, 1.12) |  | 0.99 | (0.90,1.11) |  |  | 0.91 | (0.75, 1.10) |  | 0.96 | (0.81, 1.13) |
| **rs1205** |  | |  |  |  |  |  |  |  |  |  |  |  |  |
| TT | 22/49 | | 1.00 | Reference | 26/49 | 1.00 | Reference |  | 13/33 | 1.00 | Reference | 15/33 | 1.00 | Reference |
| CT | 78/241 | | 0.45 | (0.26, 0.79) | 103/241 | 0.46 | (0.28, 0.76) |  | 31/106 | 0.88 | (0.42, 1.88) | 41/106 | 1 | (0.50, 2.00) |
| CC | 88/265 | | 0.52 | (0.30, 0.91) | 121/265 | 0.56 | (0.35, 0.92) |  | 33/122 | 0.78 | (0.36, 1.69) | 40/122 | 0.84 | (0.42, 1.69) |
| p-trend |  | |  | 0.34 |  |  | 0.56 |  |  |  | 0.52 |  |  | 0.50 |
| per C allele |  | | 0.88 | (0.68, 1.14) |  | 0.94 | (0.75, 1.17) |  |  | 0.89 | (0.61, 1.28) |  | 0.89 | (0.65, 1.24) |
| TT vs CT+CC (analogous to other publications) | | | 2.05 | (1.20, 3.51) |  | 1.94 | (1.22, 3.10) |  |  | 1.20 | (0.59, 2.43) |  | 1.09 | (0.57, 2.10) |
| **rs1800947** | |  |  |  |  |  |  |  |  |  |  |  |  |  |
| CG/GG | | 25/77 | 1.00 | Reference | 33/77 | 1.00 | Reference |  | 10/31 | 1.00 | Reference | 10/31 | 1.00 | Reference |
| CC | | 163/478 | 1.06 | (0.65, 1.73) | 217/478 | 0.97 | (0.64, 1.46) |  | 67/229 | 0.80 | (0.35, 1.81) | 86/229 | 1.17 | (0.54, 2.56) |
| p-trend | |  |  | 0.65 |  |  | 0.09 |  |  |  | 0.45 |  |  | 0.86 |
| per C allele | |  | 1.12 | (0.70, 1.78) |  | 1.03 | (0.70, 1.51) |  |  | 0.75 | (0.36, 1.56) |  | 1.06 | (0.52, 2.17) |
| **rs1130864** | |  |  |  |  |  |  |  |  |  |  |  |  |  |
| GG | | 84/246 | 1.00 | Reference | 109/246 | 1.00 | Reference |  | 38/116 | 1.00 | Reference | 47/116 | 1.00 | Reference |
| GA | | 82/245 | 0.93 | (0.66, 1.30) | 107/245 | 0.92 | (0.68, 1.22) |  | 33/118 | 0.91 | (0.51, 1.60) | 41/118 | 0.85 | (0.52, 1.40) |
| AA | | 22/64 | 1.20 | (0.70, 2.04) | 34/64 | 1.41 | (0.92, 2.17) |  | 6/27 | 0.55 | (0.20, 1.50) | 8/27 | 0.62 | (0.26, 1.45) |
| p-trend | |  |  | 0.79 |  |  | 0.37 |  |  |  | 0.29 |  |  | 0.25 |
| per A allele | |  | 1.03 | (0.81, 1.33) |  | 1.10 | (0.89, 1.35) |  |  | 0.80 | (0.54, 1.20) |  | 0.81 | (0.57, 1.16) |
| **rs3093077** | |  |  |  |  |  |  |  |  |  |  |  |  |  |
| AA | | 164/481 | 1.00 | Reference | 220/481 | 1.00 | Reference |  | 72/234 | 1.00 | Reference | 88/234 | 1.00 | Reference |
| AC/CC | | 24/74 | 0.89 | (0.55, 1.44) | 30/74 | 0.80 | (0.53, 1.21) |  | 5/27 | 0.73 | (0.27, 1.96) | 8/27 | 0.79 | (0.35, 1.77) |
| p-trend | |  |  | 0.70 |  |  | 0.33 |  |  |  | 0.54 |  |  | 0.57 |
| per C allele | |  | 0.91 | (0.58, 1.44) |  | 0.82 | (0.55, 1.22) |  |  | 0.73 | (0.27, 1.96) |  | 0.79 | (0.35, 1.77) |

Adjusted for age at diagnosis (in years as a continuous variable), sex, tumor stage (I-IV, unknown), grade of tumor differentiation (well differentiated, moderately differentiated, poorly differentiated, or unknown), location of primary tumor (colon or rectum) and year of diagnosis

Supplemental table 6. Association between genetic predisposition to higher CRP concentrations and colorectal cancer outcome, models stratified by stage

|  | **Stage I or II** | | | | | | |  | **Stage III or IV** | | | | | |
| --- | --- | --- | --- | --- | --- | --- | --- | --- | --- | --- | --- | --- | --- | --- |
|  | CRC mortality | | | | All-cause mortality | | |  | CRC mortality | | | All-cause mortality | | |
|  | N event/N Total | | HR | (95% CI) | N event/N Total | HR | (95% CI) |  | N event/N Total | HR | (95% CI) | N event/N Total | HR | (95% CI) |
| **Weighted CRP-score** |  | |  |  |  |  |  |  |  |  |  |  |  |  |
| Tertile 1 | 31/133 | | 1 | Reference | 40/133 | 1 | Reference |  | 54/92 | 1 | Reference | 67/92 | 1 | Reference |
| Tertile 2 | 19/124 | | 0.61 | (0.32, 1.16) | 29/124 | 0.7 | (0.41, 1.18) |  | 40/93 | 0.69 | (0.44, 1.08) | 52/93 | 0.65 | (0.43, 0.98) |
| Tertile 3 | 23/133 | | 0.66 | (0.37, 1.21) | 33/133 | 0.71 | (0.43, 1.18) |  | 59/110 | 0.88 | (0.58, 1.34) | 68/110 | 0.86 | (0.58, 1.26) |
| p-trend |  | |  | 0.14 |  |  | 0.16 |  |  |  | 0.48 |  |  | 0.33 |
| per score unit |  | | 0.85 | (0.71, 1.01) |  | 0.91 | (0.79, 1.06) |  |  | 0.98 | (0.85, 1.13) |  | 0.97 | (0.85, 1.10) |
| **rs1205** |  | |  |  |  |  |  |  |  |  |  |  |  |  |
| TT | 10/35 | | 1 | Reference | 11/35 | 1.00 | Reference |  | 18/28 | 1.00 | Reference | 21/28 | 1.00 | Reference |
| CT | 34/174 | | 0.48 | (0.22, 1.03) | 49/174 | 0.69 | (0.35, 1.37) |  | 62/124 | 0.53 | (0.28, 1.00) | 80/124 | 0.61 | (0.34, 1.09) |
| CC | 29/181 | | 0.38 | (0.17, 0.83) | 42/181 | 0.52 | (0.26, 1.05) |  | 73/143 | 0.58 | (0.31, 1.09) | 86/143 | 0.67 | (0.37, 1.18) |
| p-trend |  | |  | 0.04 |  |  | 0.05 |  |  |  | 0.40 |  |  | 0.51 |
| per C allele |  | | 0.66 | (0.45, 0.97) |  | 0.73 | (0.53, 1.00) |  |  | 0.89 | (0.67, 1.18) |  | 0.92 | (0.71, 1.19) |
| TT vs CT+CC (analogous to other publications) | | | 2.32 | (1.12, 4.80) |  | 1.65 | (0.86, 3.19) |  |  | 1.79 | (0.98, 3.29) |  | 1.57 | (0.90, 2.72) |
| **rs1800947** | |  |  |  |  |  |  |  |  |  |  |  |  |  |
| CG/GG | | 11/52 | 1 | Reference | 13/52 | 1.00 | Reference |  | 19/34 | 1.00 | Reference | 24/34 | 1.00 | Reference |
| CC | | 62/338 | 0.74 | (0.37, 1.48) | 89/338 | 0.99 | (0.54, 1.82) |  | 134/261 | 0.98 | (0.56, 1.73) | 163/261 | 0.86 | (0.52, 1.43) |
| p-trend | |  |  | 0.40 |  |  | 0.92 |  |  |  | 0.96 |  |  | 0.57 |
| per C allele | |  | 0.77 | (0.42, 1.41) |  | 1.03 | (0.60, 1.75) |  |  | 0.98 | (0.56, 1.73) |  | 0.86 | (0.52, 1.43) |
| **rs1130864** | |  |  |  |  |  |  |  |  |  |  |  |  |  |
| GG | | 35/177 | 1 | Reference | 46/177 | 1 | Reference |  | 64/122 | 1 | Reference | 81/122 | 1 | Reference |
| GA | | 34/171 | 0.99 | (0.58, 1.69) | 45/171 | 1.08 | (0.69, 1.70) |  | 69/138 | 0.85 | (0.58, 1.23) | 82/138 | 0.83 | (0.59, 1.17) |
| AA | | 4/42 | 0.44 | (0.15, 1.31) | 11/42 | 0.84 | (0.41, 1.69) |  | 20/35 | 1.20 | (0.67, 2.15) | 24/35 | 1.24 | (0.73, 2.10) |
| p-trend | |  |  | 0.26 |  |  | 0.80 |  |  |  | 0.95 |  |  | 0.93 |
| per A allele | |  | 0.80 | (0.53, 1.18) |  | 0.96 | (0.71, 1.31) |  |  | 1.01 | (0.76, 1.33) |  | 1.01 | (0.78, 1.30) |
| **rs3093077** | |  |  |  |  |  |  |  |  |  |  |  |  |  |
| AA | | 66/344 | 1 | Reference | 94/344 | 1.00 | Reference |  | 137/261 | 1.00 | Reference | 167/261 | 1.00 | Reference |
| AC/CC | | 7/46 | 0.82 | (0.37, 1.82) | 8/46 | 0.58 | (0.28, 1.22) |  | 16/34 | 0.87 | (0.49, 1.53) | 20/34 | 0.91 | (0.55, 1.50) |
| p-trend | |  |  | 0.86 |  |  | 0.25 |  |  |  | 0.63 |  |  | 0.70 |
| per C allele | |  | 0.94 | (0.45, 1.96) |  | 0.66 | (0.33, 1.33) |  |  | 0.87 | (0.49, 1.53) |  | 0.91 | (0.55, 1.50) |

Adjusted for age at diagnosis (in year as a continuous variable), sex, grade of tumor differentiation (well differentiated, moderately differentiated, poorly differentiated, or unknown), location of primary tumor (colon or rectum), and year of diagnosis
